# Patients’ perception and satisfaction on quality of laboratory malaria diagnostic service in Amhara Regional State, North West Ethiopia

**DOI:** 10.1186/s12936-015-0756-6

**Published:** 2015-06-11

**Authors:** Agajie Likie Bogale, Habtamu Belay Kassa, Jemal Haidar Ali

**Affiliations:** Addis Ababa City Administration Health Bureau, Health Research Laboratory, Addis Ababa, Ethiopia; Microbiology, Immunology and Parasitology Department, Addis Ababa University, Addis Ababa, Ethiopia; School of Public Health, Addis Ababa University, P.O. Box 27285/1000, Addis Ababa, Ethiopia

## Abstract

**Background:**

The most effective strategies in the fight against malaria are to correctly diagnose and timely treat the illness. A diagnosis based on clinical symptoms alone is subjected to misuse of anti-malarial drugs, increased costs to the health services, patient dissatisfaction and also contributes to an increase in non-malaria morbidity and mortality. Among others, inappropriate perception and inadequate satisfaction of patients are significant challenges reported to affect the quality of laboratory malaria diagnostic services.

**Methods:**

A facility-based, cross-sectional study was conducted from November to December 2013 among 300 patients. Their level of satisfaction was measured using both pre-tested structured and open ended questionnaires. A 5-point Likert scales and their weighted average were used to categorize satisfaction level of the patients. Data were entered in Epi-Info version 3.5.3 and analysed using SPSS version 20. Chi-square test was used to see the association between the outcome variable and independent and the strength of the association was identified using odds ratio in the binary logistic regression. In addition the open ended questionnaire findings were coded and analysed thematically.

**Results:**

Over half (52.6 %) of the patients were satisfied with the malaria diagnostic service with a 98.7 % response rate. The majority (89.3 %) of patients perceived they were well diagnosed in facing fever upon giving blood for laboratory malaria diagnosis within 30 min waiting time in most (62.5 %) of the patients. Ethnicity, residence, knowing malaria diagnosis after consulting clinician, and time period to receive malaria result were the independent predictors for patient satisfaction (*p* < 0.05). The open ended questionnaire responses also revealed providing precise laboratory result timely, availability of the right treatment, presence of health professionals performing the laboratory test upon request in the health facility were among the major enabling factors for patients’ satisfaction.

**Conclusion:**

The observed level of satisfaction in the current study though encouraging when compared with some previous studies conducted in eastern Ethiopia on general laboratory services, still it requires scale-up in the enhancement of malaria laboratory diagnostic service in the fight against malaria.

## Background

Malaria is a major global health problem [1]. According to the latest estimates, 198 million cases of malaria occurred globally in 2013 and the disease led to 584, 000 deaths. The majority (78 %) of all deaths encountered in children under five [2].

Ethiopia is one of the most malaria epidemic-prone countries in Africa. Rates of morbidity and mortality increase dramatically during epidemics. Approximately 52 million people (68 %) live in malaria risk areas in Ethiopia, primarily at altitudes below 2000 m [3]. Correct diagnosis and effective treatment are among the main strategies in the fight against malaria. A diagnosis based on clinical symptoms alone has very low specificity and contributes to an increase in non-malaria morbidity and mortality, the misuse of anti-malarial drugs, increased costs to the health services and patient dissatisfaction [1].

Satisfaction has been defined as a consumer’s emotional feelings about a specific consumption experience [4]. Patient satisfaction is the extent to which the patients feel that their needs and expectations are being met by the service provided [5, 6]. It is as important as other clinical health measures and is a primary means of measuring the effectiveness of health care delivery [7, 8]. According to Rasheed et al*.*, patient’s dissatisfaction emanates from poor quality microscopy, particularly at the peripheral level and delays in providing results to clinical staff affecting quality of malaria laboratory diagnosis [8]. Other than this, overcrowding, delay in consultation, lack of proper guidance also leads to patient dissatisfaction [5, 9]. Importantly, patients’ perceptions is also, an important factor in utilizing the available healthcare delivery services, seem to have been largely ignored by health care managers in developing countries [9]. Despite the fact that, it is one of the established yardsticks to measure success in the overall health system [4, 6]. The prevailing inappropriate perception and inadequate satisfactions of health service providers and users on quality of laboratory service present which is a means to manage patient’s response to treatment as well as monitor disease trends in particular [10] however, pauses a significant challenge in the utilization of the services [10]. Therefore, this study was done to assess patient’s perceptions and satisfaction, particularly on the laboratory services which is a basis for good clinical diagnosis and patient management and provide users/care takers and stakeholders evidence based information on the benefit of quality laboratory malaria diagnostic service contextually.

## Methods

### Study area and population

A health facility based cross sectional study was conducted in Awi zone, Amhara region, North West Ethiopia from November to December 2013. The zone is 420 km north of Addis Ababa with astronomical location is 10° 53′ North Latitude and 36° 56′ East Longitude) and it is among the 11 zones of the Regional State with 11 districts (*Woredas*). It is a home for 1,119,555 people of whom 560,129 were males and 559,426 females. The predominant ethnic groupings in the zone are Awi and Amhara [11]. Since malaria is endemic and reported as a major killer in developing countries [12], the site was deliberately selected for the assessment of quality of laboratory malaria diagnostic services. Among the 11 districts of the zone, six of them were malaria endemic and of these, three districts were selected randomly. A total of 12 health centres available in the selected districts were included in the actual study.

### Ethical considerations

Ethical clearance was obtained from Departmental Research and Ethics Review Committee of Medical Laboratory Science department; Addis Ababa University and permission was obtained from Amhara Regional Health Bureau. The objectives of the study were explained to the study participants and informed consent was obtained before interviewing each participant. All the information obtained from the study participants was kept confidential, names or personal identifiers were not included and identification of each participant was only possible through numerical codes.

### Sample size determination, data entry, processing and analysis

The required sample size was determined by using single population formula by considering the following assumptions: Proportion of 90 % (considering that patient satisfaction on quality laboratory malaria diagnosis and service delivery conducted in previous studies in Tanzania) [10]. Level of significance = 0.05 with Marginal of error (d) = 5 %, Non-response rate = 10 % and design effect of 2. Hence, the minimum required sample size was 304 patients.

A total of 304 were drawn from the twelfth health centres namely Agew Gimjabet, Wumbry- Wundigy, Chagni, Gissa, Azena, Ayehu, Degera, Buya, Gumdry, Chara, Affessa and Abadra and enrolled for the assessment.

Adding an estimated 10.0 % for the non-response rate and a design effect of 2, a total of 304 patients made up the sample size, estimated based on an absolute precision of 5.0 % with a 95 % confidence level, and 90.0 % patient satisfaction. The numbers of patients enrolled in each health facility were 25–25 on average.

Pre-tested interviewer-administered structured as well as open ended questionnaires were used for the data collection. The important variables included in the questionnaire were the level of satisfaction (measured by Easy to access the service, Waiting time for laboratory service, Health professionals work ethics, Encouraged to ask any information, Phlebotomy services for diagnostic purposes, Missing of laboratory malaria results, Perception about quality of laboratory result, Willingness to conduct laboratory investigation, Staff language to respond to patient’s request, concern and prescription related) and socio-demographic variables. Five data collectors fluent in the local languages of the study zone (two laboratory professionals and three clinical nurses) with relevant experience were recruited and trained for 2 days on the method of the data collection. The training addressed issues such as the content of the questionnaire, basic interviewing skills, and filling out of the questionnaire. A 5-point Likert scale ranging from poor (1 point) to excellent (5 points) for few items was used to asses all the items.

Data were edited manually initially, and then entered and organized using Epi Info version 3.5.3 and exported to SPSS version 20 for descriptive and inferential analyses. The results are presented in percentages and graphs where appropriate. The mean Likert scale score or weighted average was used to categorize the satisfaction level as satisfied when the score is ≥ the mean score while the value below the mean score was taken as dissatisfied. The mean rating score for each item was calculated by multiplying the number of answers or responses in each category by its rating value (1 to 5), obtaining a sum and dividing by the total number of responses for that item; that is overall rate of satisfaction by Likert scale was calculated as (No. of excellent rating ×5) + (No. of very good rating ×4) + (No. of good rating ×3) + (No. of fair rating ×2) + (No. of poor rating ×1) divided by the total number of responses for the specific item [13, 14].

Binary logistic regression was employed to examine the associations between the outcome variables (perception and satisfaction towards laboratory malaria diagnostic services) with the various independent factors (socio-demographic and other important variables mentioned above), and the results are presented using odds ratios (ORs) and confidence intervals (95 % CI).

To ascertain the association between the dependent variables and the explanatory variables, simultaneously controlling for the aforementioned explanatory variables, (All socio-demographic characteristics and other covariates associated in univariate with *p* < 0.2 were used and entered) stepwise logistic regression was applied and adjusted odds ratios (AORs) and confidence intervals (95 % CI) were constituted. In all analyses, *P* < 0.05 was considered to be statistically significant. The open ended questionnaire findings were coded and analysed thematically.

## Results

A total of 300 patients participated in the study with a response rate of 98.7 %. Nearly half of the respondents (46.0 %) were in the age range of 18–25 and the mean age was 31 ± 13 years. Over half (53.5 %) were males. Most (69.4 %) were married, and were of Awi ethnicity. The majority were from rural (84.9 %), over half (53 %) were farmers and less than half (45 %) had no formal education (Table 1).

**Table 1:** Socio-demographic characteristics of respondents’ in Awi zone, Amhara Regional State, North West Ethiopia, 2013 (*n* = 300)

Respondents’ characteristics	Frequency	Percent
Age (in years)		
18–25	138	46.0
26–35	90	30.0
36–45	36	12.0
46–55	15	5.0
>55	21	7.0
Mean (±SD)	31 (±13)	
Sex		
Male	160	53.5
Female	139	46.5
Marital status		
Single	85	28.9
Married	204	69.4
Divorced	2	0.7
Widowed	3	1.0
Patient’s ethnicity		
Awi	196	65.3
Amhara	103	34.3
Other	1	0.3
Patient work area		
Rural	254	84.9
Urban	45	15.1
Employment		
Farmer	159	53.0
Merchant	26	8.7
Government employee	34	11.3
Non-government employee	2	0.7
Other	79	26.3
Educational		
No formal education	135	45.0
Primary education	84	28.0
Secondary education	47	15.7
College/university	33	11.0
Other	1	0.3

### Patients’ diagnosis and their various responses on malaria

About two-thirds (64.7 %) of the respondents knew about their diagnosis from their consulting clinicians, 46 (15.3 %) diagnosed themselves, 15 (5.0 %) from their consulting laboratory personnel, and 36 (12.0 %) knew from their consulting clinician and laboratory personnel. The majority (89.3 %) perceived they were diagnosed properly as having malaria when they had fever by laboratory while the remaining 7 (2.3 %) when bought drug from pharmacy, 4 (1.3 %) clinically and the rest 21 (7.0 %) through the combination of the aforementioned processes.

Regarding waiting time to obtain the laboratory result, most (62.5 %) of them received the result within 30 min, 84 (28.1 %) within 1 h, and 29 (9.4 %) after 1 h. Over two third (67.3 %) felt the waiting time was acceptable and the frequency of care given during malaria illness, over half (54.2 %) perceived they were supported all the time and the rest 103 (34.4 %) and 34 (11.3 %) stated the care given was most of the time and sometimes, respectively. The majority’s (87.6 %) of providers were concerned towards their work ethics to patient’s management (Table 2).

**Table 2:** Patients’ diagnosis and their various responses on malaria, in Awi zone, Amhara Regional State, North West Ethiopia, 2013 (*n* = 300)

Respondents’ characteristics	Frequency	Percent
Diagnosed the illness		
Clinician	194	64.7
Self	46	15.3
Lab tech	15	5.0
Lab tech and Clinician	36	12.0
Perceived to be diagnosed properly by		
Lab tech	266	89.3
Clinician	4	1.3
Pharmacy	7	2.3
Combined	21	7.0
Waiting time		
Within 30 min	187	62.5
30–60	84	28.1
>60 min	29	9.4
Frequency of care given when had malaria		
All the time	162	54.2
Most of the time	103	34.4
Some times	33	11.0
Never	1	0.3
Work ethics of providers to patients		
Very concerned	123	41.1
Concerned	139	46.5
Less concerned	36	12.0

The weighted average for the various perception and satisfaction items assessed is depicted in Table 3. Service accessibility (4.01), waiting time (3.88), respectfulness (4.01), encouragement to ask questions (3.95), phlebotomy service (4.1), test availability (4.2), perception about quality laboratory result (3.92), willingness to conduct laboratory investigation (4.09), availability of service providers (4.19), staff language to communicate (4.67), response to the requested problem (4.25), and explanation about prescribed drug (4.47) suggesting that all items were within acceptable range. The highest mean rating score was found to be staff language to communicate (4.67) and the lowest mean rating score was waiting time for laboratory service (3.88). The cut-off point for perception and satisfaction for the aforementioned items was 48. The proportion of respondents who had more than the mean score was 52.6 % indicating that more than half were satisfied.

**Table 3:** Response to different perception and satisfaction questions by patients from twelve health centers in Awi zone, Amhara Regional State, North Western Ethiopia, 2013 (*n* = 300)

Respondent’s perception and satisfaction	Excellent	Very good	Good	Fair	Poor	Weighted average	Unweighted average (mean ± SD)
	f (%)	f (%)	f (%)	f (%)	f (%)		
Easy to access the service	110 (36.7)	91 (30.3)	91 (30.3)	7 (2.3)	1 (0.3)	4.01	1.99 ± 0.89
Waiting time for lab service	104 (34.7)	79 (26.3)	95 (31.7)	21 (7.0)	1 (0.3)	3.88	2.12 ± 0.98
Health professionals respectfulness^a^	108 (36.5)	101 (34.1)	74 (25.0)	9 (3.0)	4 (1.4)	4.01	1.99 ± 0.93
Encouraged to ask any information^a^	106 (40.8)	54 (20.8)	85 (32.7)	10 (3.8)	5 (1.9)	3.95	2.05 ± 1.03
Phlebotomy service for malaria examination^a^	100 (33.6)	131 (44.0)	66 (22.1)	−	1 (0.3)	4.10	1.9 ± 0.76
Availability of lab malaria results/not missing^a^	105 (35.4)	158 (53.2)	24 (8.1)	8 (2.7)	2 (0.7)	4.2	1.8 ± 0.75
Perception about quality of lab result^a^	95 (31.9)	93 (31.2)	104 (34.9)	4 (1.3)	2 (0.7)	3.92	2.08 ± 0.88
Willingness to conduct lab investigation ^a^	107 (35.7)	115 (38.3)	76 (25.3)	1 (0.3)	1 (0.3)	4.09	1.91 ± 0.81
Punctuality of service providers during working hours	114 (38.0)	138 (46.0)	40 (13.3)	7 (2.3)	1 (0.3)	4.19	1.81 ± 0.78
Staff language to communicate	224 (74.7)	57 (19.0)	15 (5.0)	3 (1.0)	1 (0.3)	4.67	1.33 ± 0.65
The response to your request, and problems by lab personnel^a^	134 (44.8)	112 (37.5)	51 (17.1)	1 (0.3)	1 (0.3)	4.25	1.74 ± 0.77
Explanation about prescribed malaria drug	160 (53.3)	123 (41.0)	15 (5.0)	1 (0.3)	1 (0.3)	4.47	1.53 ± 0.64

“f” indicates number of respondents for single variable

*SD* standard deviation

^a^sample size do not add up to 300 because of illogical response

Although age (*p* = 0.032), sex (*p* = 0.007), ethnicity (*p* = 0.001), rural residency (*P* = 0.001), education (*p* = 0.002), employment (*p* = 0.005), knowledge of malaria (*p* = 0.001), and waiting time to receive laboratory result (*p* = 0.001) were crudely associated with the perception and satisfaction, only ethnicity, residency, knowledge of malaria and waiting time (*p* < 0.05) retained their significant associations. Being Amhara was 75.5 % (AOR = 0.24, 95 % CI = 0.10–0.55) less likely to be satisfied than Awi. Rural residency was almost five times more likely satisfied (AOR = 4.89, 95%CI = 1.07–22.28) in malaria diagnostic service than urban (town) residents. Those patients who knew malaria diagnosis after consulting clinicians were 3.3 times more likely satisfied (AOR = 3.32, 95%CI = 1.11–9.88) than those who knew by themselves, and those patients who obtained their result from 30 min to 1 h were 87.7 % (AOR = 0.12, 95%CI = 0.05–0.28) less likely to be satisfied than those received within 30 min (Table 4).

**Table 4:** Univariate and Multivariate analysis for predictors of satisfaction towards patient respondents in selected health centers Awi Zone, Amhara Regional State, North West Ethiopia, 2013 (*n* = 300)

Characteristics	Outcome		COR (95%CI)	AOR (95%CI)
	Satisfied (≥48) Freq (%)	Dissatisfied (≤47) Freq (%)		
Age				
18–25	57 (48.7)	60 (52.3)	1	
26–35	37 (52.9)	33 (47.1)	1.26 (1.02–1.55)*	1.30 (0.95–1.78)
36–45	15 (44.1)	19 (55.9)		
46–55	9 (69.2)	4 (30.8)		
>55	15 (78.9)	4 (21.1)		
Sex				
Female	48 (42.9)	64 (57.1)	1	1
Male	84 (60)	56 (40)	2.0 (1.20–3.31)*	1.35 (0.64–2.86)
Ethnicity				
Awi	114 (67.5)	55 (32.5)	1	1
Amhara	18 (21.7)	65 (78.3)	0.13 (0.07–0.24)*	0.24 (0.11–0.55)*
Work area				
Urban	4 (12.5)	28 (87.5)	1	1
Rural	129 (58.6)	91 (41.4)	9.92 (3.36–29.26)*	4.89 (1.07–22.28)*
Educational level				
Illiterate	54 (50)	54 (50)	1	1
Primary education	35 (46.1)	41 (53.9)	0.85 (0.47–1.54)	0.71 (0.24–2.14)
Secondary education	19 (48.7)	20 (51.3)	0.95 (0.45–1.97)	0.63 (0.18–2.18)
College/university	25 (83.3)	5 (16.7)	5.0 (1.78–14.03)*	2.99 (0.36–24.88)
Employment				
Farmer	68 (51.5)	64 (48.5)	1	1
Merchant	8 (36.4)	14 (63.6)	0.53 (0.21–1.36)	1.62 (0.39–6.72)
Government employee	25 (80.6)	6 (19.4)	3.92 (1.51–10.18)*	4.01 (0.616–26.11)
Non-government employee	1 (50)	1 (50)	0.94 (0.05–15.36)	0.99 (0.01–489.99)
Other	31 (47)	35 (53)	0.83 (0.46–1.50)	0.96 (0.28–3.30)
Knowing malaria diagnosis				
By your selves	7 (19.4)	29 (80.6)	1	1
After consulting clinician	100 (61.3)	63 (38.7)	6.576 (2.718–15.910)*	3.32 (1.11–9.88)*
After consulting lab personnel	6 (40)	9 (60)	2.76 (0.74–10.36)	1.2 (0.23–6.65)
Waiting time to receive malaria lab result				
Within 30 min	119 (72.6)	45 (27.4)	1	1
30 min–1 h	14 (19.7)	57 (80.3)	0.09 (0.05–0.18)	0.12 (0.05–0.28)*
After 1 h	0	10 (100)	0.001 (0.001)	0.001 (0.001)

* *p* < 0.05

According to the open ended responses, the reason for satisfaction was stated to be the use of confirmatory laboratory examination and the timeliness of the diagnosis. Other important information stated for their satisfactions were laboratory professional’s respect and work ethics including the presence of health professionals in the health facility when needed. Very few respondents however, were dissatisfied because of shortage of essential materials for the diagnostic service and inconsistencies in laboratory results. One respondent said that “*for me satisfaction means when I am properly diagnosed and get cured*” another patient added “*I want to hear about my disease from the laboratory personnel who performs the diagnosis*” and three others said that “*when we have the symptoms of malaria, service providers tell us to have no malaria which when checked at private clinic we were told positive for malaria. Same respondents mentioned such experiences are due to lack of sufficient time during examination, misconduct and unavailability of malaria drugs and suggested that; laboratory diagnosis should be provided always prior to ordering drug and malaria drug should be accessible in health centers since it is unaffordable to buy from private pharmacy and there is unwanted transport cost because of their inaccessibility as a concluding remark.*

## Discussion

The study is first of its kind to assess the perception and satisfaction of patients towards laboratory malaria diagnosis service in selected Health facilities of Awi zone, North West Ethiopia, and revealed an overall single item based patient satisfaction rate of 90.7 % which is somewhat similar with the findings of Derua et al*.* who reported 90.0 % in Tanzania [10]. When compared with available figures reported on satisfaction for the general and ART laboratory services from eastern Ethiopia and Addis Ababa, respectively, the rate of satisfaction in the current study was higher than the study conducted in eastern Ethiopia on general laboratory service [15] and study done in public hospitals in Addis Ababa on ART service [16] probably due to population difference.

In this study, the indigenous ethnicities, Awi, were more satisfied than Amhara unlike a study conducted in Trinidad and Tobago which indicated that ethnicity had no influence on the degree of patient’s perception and satisfaction towards health care service [13]. This is probably due to the fact that the majority of the respondents were Awis, and Amharas were immigrants who had higher expectations. Lending support to this finding, patients from rural residence were more satisfied in the service than urban residents and the finding is inconsistent with the study conducted in eastern Ethiopia where they observed urban residents were more satisfied than their rural counterparts [15], probably due to population differences.

Although the majority of respondents in this study had no formal schooling and were working in rural area, surprisingly almost all perceived that they were diagnosed correctly when tested for malaria (89.3 %) and were satisfied with the service since the diagnosis was confirmed through laboratory and most importantly saved them from unnecessary medications. This finding was also reflected in the perception assessment and consistent with the southwest Nigerian [17], Ghanaian [18] and North Eastern Tanzanian [19] studies.

This study also depicted that, waiting time to obtain malaria diagnostic result had a significant association with degree of satisfaction and it is concurs with the study conducted on ART laboratory service [16] in Addis Ababa. Nonetheless, the study conducted in Trinidad and Tobago had showed that waiting time had no influence on satisfaction [13] probably due to different study settings.

In this study, knowing malaria diagnosis after consulting clinicians rather than taking self treatment provided more satisfaction score for patients and was found to be 52.6 %. This finding was in agreement with some previous studies conducted in North Eastern Tanzania which indicated that knowing the malaria test result was significantly associated with expressing satisfaction at interview [19].

In conclusion, the majority of patients in this study perceived that they were well diagnosed in facing fever when they provide blood for laboratory examination and satisfied when further laboratory investigations were conducted suggesting the level of satisfaction is encouraging. Patients’ ethnicity, residence, knowing malaria diagnostic result after consulting clinicians, and waiting time to receive malaria laboratory results were important deriving factors for satisfaction. Therefore, to maintain the present findings, scale-up in the enhancement of malaria laboratory diagnostic service that includes the aforementioned deriving factors in the fight against malaria are recommended.

### Strength and limitations

This study was done during the malaria season and it is the first of its kind, using structured and open ended questionnaires including suggestions from the respondents as a means to scale up in the enhancement of malaria laboratory diagnostic service. Nonetheless, it was not easy to measure the temporal relationship since both exposure and outcome variables were collected simultaneously and this was a limitation of the study. The inclusion of healthcare providers would have revealed some other deriving factors related to patients’ satisfaction.

## References

[1] Pfeiffer K, Some F, Müller O, Sie A, Kouyate B, Haefeli WE (2008). Clinical diagnosis of malaria and the risk of chloroquine self-medication in rural health centers in Burkina Faso. Trop Med Int Health.

[2] WHO (2014). World malaria report, 2014.

[3] Federal Democratic Republic of Ethiopia Ministry of Health (FMOH) (2012). National malaria guidelines, Addis Ababa-Ethiopia.

[4] Sodani PR, Sharma K (2011). Assessing patient satisfaction for investigative services at public hospitals to improve quality of services. Nat J Com Med.

[5] Iliyasu Z, Abubakar IS, Lawan UM, Gajida AU (2010). Patients’ satisfaction with services obtained from Aminu Kano Teaching Hospital, Kano, Northern Nigeria. Nigerian J Clin Pract.

[6] Arshad AS, Shamila H, Jabeen R, Fazli A (2012). Measuring patient satisfaction, a cross sectional study to improve quality of care at a tertiary care hospital. Healthline.

[7] Kumari R, Idris MZ, Bhushan V, Khanna A, Agarwal M, Singh SK (2009). Study on patient satisfaction in the government allopathic health facilities of Lucknow district, India. Indian J Community Med.

[8] Rasheed N, Arya S, Acharya A, Khandekar J (2012). Client satisfaction and perceptions about quality of health care at primary health center of Delhi, India. Indian J Community Health.

[9] Jenkinson C, Coulter A, Bruster S, Richards N, Chandola T (2002). Patient’s experiences and satisfaction with healthcare: results of a questionnaire study of specific aspects of care. Qual Saf Health Care.

[10] Derua YA, Ishengoma DRS, Rwegoshora RT, Tenu F, Massaga JJ, Mboera LEG (2011). Users’ and health service providers’ perception on quality of laboratory diagnosis in Tanzania. Malar J.

[11] Central Statistical Agency (CSA) (2007). Census conducted in Ethiopia.

[12] Baltzell K, Elfving K, Shakely D, Ali AS, Msellem M, Gulati S (2013). Febrile illness management in children under five years of age: a qualitative pilot study on primary health care workers’ practices in Zanzibar. Malar J.

[13] Singh H, Haqq ED, Mustapha N (1999). Patients’ perception and satisfaction with health care professionals at primary care facilities in Trinidad and Tobago. Bull World Health Organ.

[14] Taylor-Powell E (1989). Program development and evaluation, analyzing quantitative data.

[15] Teklemariam Z, Mekonnen A, Kedir H, Kabew G (2013). Clients and clinician satisfaction with laboratory services at selected government hospitals in eastern Ethiopia. BMC Res Notes.

[16] Mindaye T, Taye B (2012). Patient’s satisfaction with laboratory services at antiretroviral therapy clinics in public hospitals, Addis Ababa. Ethiopia BMC Res Notes.

[17] Iwelunmor J, Airhihenbuwa CO, King G, Adedokun A (2013). Contextualizing child malaria diagnosis and treatment practices at an outpatient clinic in southwest Nigeria: a qualitative study. ISRN, Infectious Diseases.

[18] Ansah EK, Reynolds J, Akanpigbiam S, Whitty CJM, Chandler CIR (2013). “Even if the test result is negative, they should be able to tell us what is wrong with us”: a qualitative study of patient expectations of rapid diagnostic tests for malaria. Malar J.

[19] Chandler R, Mwangi R, Olomi R, Mbakilwa H, Whitty C, Reyburn H (2008). Malaria over diagnosis: is patient pressure the problem?. Health Policy Plan.

